# The Adult Inpatient eHealth Literacy Scale (AIPeHLS): Development and Validation Study

**DOI:** 10.2196/75657

**Published:** 2025-10-14

**Authors:** Xinyu Feng, Vivian Hui, Jing Jiang, Mengyuan Liu, Yinglan Li, Lingyun Tian

**Affiliations:** 1 Centre for Smart Health, School of Nursing, Hong Kong Polytechnic University, Kowloon, China (Hong Kong); 2 Xiangya School of Nursing, Central South University, Changsha, Hunan, China; 3 Health and Community Systems, School of Nursing, University of Pittsburgh, Pennsylvania, United States; 4 Teaching and Research Section of Clinical Nursing, Xiangya Hospital, Central South University, Changsha, Hunan, China; 5 National Clinical Research Center of Geriatric Disorder, Xiangya Hospital, Central South University, Changsha, Hunan, China; 6 Department of Health Management Center, The First Affiliated Hospital of USTC, Division of Life Sciences and Medicine, University of Science and Technology of China, No.17, Lujiang Road, Hefei, Anhui, 230001, China, 86 18225853895; 7 Department of Nursing, The First Affiliated Hospital of USTC, Division of Life Sciences and Medicine, University of Science and Technology of China, Hefei, Anhui, China

**Keywords:** eHealth literacy, inpatients, scale development, validity and reliability, health care innovation

## Abstract

**Background:**

The rapid evolution of digital health technologies, particularly within the Web 3.0 framework, has underscored eHealth literacy (eHL) as a critical competency for patients engaging with digital health care platforms. Patients in sustained hospital stays, often in vulnerable conditions, face unique challenges in using eHealth tools effectively. However, existing eHL assessment tools are insufficient to address the intricate and dynamic demands of contemporary health care systems, especially for individuals under continuous hospital care.

**Objective:**

This study aimed to develop the Adult Inpatient eHealth Literacy Scale (AIPeHLS), a comprehensive, multidimensional tool grounded in the Lily Model, to evaluate eHL among adult inpatients within the context of digital health care innovations.

**Methods:**

The development of the AIPeHLS followed a systematic, multiphase process. Initial item pool generation was informed by a literature review and then refined using the Delphi method, resulting in a preliminary set of 53 items spanning 6 dimensions of the Lily Model. The scale was refined through a pilot survey among 100 individuals requiring inpatient care, followed by item analysis and exploratory factor analysis (EFA). Validation was achieved via a cross-sectional study with 532 participants, using confirmatory factor analysis (CFA) to verify the scale structure, alongside evaluations of convergent, discriminant, criterion-related, and content validity. Reliability was assessed using Cronbach α, Omega, and split-half reliability.

**Results:**

The finalized AIPeHLS comprised 44 items across 6 dimensions: traditional literacy, information literacy, media literacy, health literacy, computer literacy, and scientific literacy, reflecting the skills necessary in the Web 3.0 context. Both EFA and CFA confirmed the 6-factor structure, demonstrating acceptable model fit indices (*χ²*=1974.654 (*df*=887), root mean square error of approximation=0.048, comparative fit index=0.957, normed fit index=0.925, and incremental fit index=0.957). The scale exhibited robust content validity, convergent and discriminant validity, criterion-related validity, and high internal consistency, with a Cronbach α of .965, Omega coefficient of 0.962, and a split-half reliability of 0.791 for the entire scale.

**Conclusions:**

The 44-item AIPeHLS was found to be a reliable and valid instrument for assessing eHL in adult inpatients in the evolving Web 3.0 context. Its comprehensive framework and strong psychometric properties make it an effective tool for health care providers to understand patients’ digital health competencies and tailor interventions accordingly. For researchers, our findings provided opportunities to explore the relationship between eHL and health outcomes, while offering valuable insights into the development of more effective eHealth interventions and policies.

## Introduction

The unprecedented advancements in information and communications technology (ICT) have significantly transformed the health care landscape, positioning digital tools as indispensable components of modern medical practice. In particular, the proliferation of generative artificial intelligence (AI) in recent years has dramatically improved access to health information. Within this context, the ability of patients to effectively engage with electronic health (eHealth) tools has become a pivotal determinant of health care outcomes. The required capabilities in this domain were initially proposed as eHealth literacy (eHL) by Norman and Skinner in 2006, defining it as “the ability to seek, find, understand, and appraise health information from electronic sources and apply the knowledge gained to addressing or solving a health problem.” [1] Subsequent research has consistently demonstrated that higher eHL is associated with superior health management, improved treatment adherence, and reduced health care costs, while insufficient eHL contributes to delayed medical interventions and poorer health outcomes [2-4].

In China, the surge of digital health initiatives, such as internet hospitals and rural health care digitization, has expanded the accessibility of health care services, with the number of internet users reaching 1.092 billion in 2024, 85% of whom are adults [5]. For instance, emerging technologies such as indoor navigation systems, AI-driven diagnostic assistance, and generative AI chatbots have shown promise in improving care delivery, reducing costs, and streamlining clinical workflows [6-8]. However, these advancements also present unique challenges, particularly for inpatients who are often in vulnerable states due to acute or chronic conditions and may lack the skills necessary to navigate complex eHealth environments. Statistics showed that 781 million adults worldwide remain illiterate, highlighting significant barriers many patients face in accessing and using health care technologies [9]. Furthermore, significant regional disparities in economic development and divide in health care resources across regions create additional barriers for rural and socioeconomically disadvantaged populations, who frequently exhibit lower eHL and limited experience with digital tools [10]. Without adequate eHL, patients may struggle to access and evaluate health information, undermining their ability to benefit from technological innovations and compromising health care outcomes.

Effective adoption of digital health services in clinical settings hinges on patients’ readiness and ability to use these technologies [11]. Inpatients often require frequent access to health information to manage their acute or complex medical conditions within a constrained timeframe. Yet, the reliability and quality of online health resources remain uncertain, and the rise of generative AI has introduced additional risks, such as hallucinated or misleading information [12]. A previous study has shown that patients increasingly rely on the internet for health-related decisions, even more frequently than they consult doctors [13]. However, they are not fully equipped with the skills to critically evaluate the credibility of digital content or to protect their personal data, with some users judging website authority solely based on superficial design elements [13]. These gaps in eHL can lead to nonadherence to medical recommendations, strained doctor–patient relationships, compromised treatment outcomes, and diminished overall patient satisfaction. Assessing eHL among inpatients is therefore essential to understanding their medical needs, providing targeted technical support, and empowering patients to actively participate in health care procedures. Such efforts can not only improve patients’ self-management capabilities and quality of life but also optimize the overall efficiency and effectiveness of health care delivery [2,14].

Despite the growing emphasis on eHL as a crucial competency in the digital health care era, existing assessment tools fail to comprehensively capture the full spectrum of eHL skills required by inpatients under the Web 3.0 era. The foundational “Lily model” of eHL, proposed by Norman and Skinner [1], identifies 6 core literacies: traditional literacy, information literacy, media literacy, health literacy, computer literacy, and scientific literacy. These literacies collectively highlight the challenges faced by individuals with limited proficiency in any one area. Importantly, eHL is not static but a dynamic, process-oriented skill that evolves alongside technological advancements and shifts in social, personal, and environmental contexts [1]. For instance, the progression of the internet from Web 1.0 (read-only) to Web 2.0 (interactive and social) and now Web 3.0 (semantic and machine-driven integration) has reshaped the demands placed on users. Web 1.0 primarily emphasized information retrieval skills, while Web 2.0 demanded interactive and collaborative abilities. Web 3.0, characterized by machine learning and data integration, requires higher-order skills such as managing personal health data, ensuring cybersecurity, and discerning trustworthy digital resources [15,16]. However, existing eHL assessment tools remain anchored in the Web 1.0 paradigm and fail to address the complex demands of contemporary digital environments. The widely used 8-item eHealth Literacy Scale (eHEALS) by Norman and Skinner [17] in 2006 has revealed significant limitations in combining clinical scenarios and reflecting competencies in the Web 3.0 context.

More recent instruments have attempted to expand the scope of eHL measurement, but notable gaps still remain. The electronic health literacy scale (e-HLS), developed by Seçkin et al [18] in 2016, primarily focused on behaviors related to information evaluation and trust but neglected foundational skills like resource access and basic technological operation. Likewise, the eHealth Literacy Questionnaire (eHLQ) by Kayser et al [19] in 2018 failed to evaluate patients’ ability to assess the credibility and authenticity of health information and lacked integration with real-world clinical settings. Despite the Transactional eHealth Literacy Instrument (TeHLI) pioneering an emphasis on interpersonal skills and the ability to apply knowledge in practice, it overlooked crucial competencies such as data tracking and adaptability to emerging technologies [20]. While Liu et al [21] introduced a tool involving privacy security, data sharing, and ownership, its focus on college students, a digitally adept group with higher baseline literacy and more frequent use of electronic devices, limited its relevance in clinical contexts and its applicability to vulnerable and less experienced populations. Van der Vaart et al [22] developed the Digital Health Literacy Instrument (DHLI), which represents a notable advancement in this domain by integrating both self-reported and performance-based measures. Unlike previous instruments, the DHLI uniquely incorporates a set of practical tasks that require respondents to demonstrate digital skills in simulated scenarios, addressing the gap between perceived and actual ability. However, its reliance on computer-based tasks may limit applicability among populations with less computer experience or those who primarily access health information via mobile devices.

Currently, there is no standardized, comprehensive instrument adapted in the new era of AI-integrated digital health care tailored to inpatients. This gap not only limits health care providers’ ability to understand patients’ eHL and facilitate their engagement with eHealth tools but also impedes the integration of innovative solutions into clinical practice. In response to these challenges, this study aimed to develop and validate the Adult Inpatient eHealth Literacy Scale (AIPeHLS), a novel assessment tool grounded in the Lily model and designed to reflect the competencies required in the Web 3.0 health care ecosystem. By addressing this gap, the AIPeHLS holds the potential to empower patients to make informed health decisions, enhance personalized care delivery, and inform the development of future digital health interventions.

## Methods

### Step 1: Development of the AIPeHLS

#### Item Pool Generation

The development of the initial item pool was guided by the Lily model, aiming to address the specific needs of inpatients and reflect application scenarios involving modern information technologies. Relevant items from validated scales in existing studies were adapted and refined to ensure consistency, clarity, and relevance to the target population.

A systematic search of both Chinese and English databases was conducted to identify relevant literature published between January 1, 2013 and April 10, 2023. Chinese databases included CNKI, Wanfang, VIP, and SinoMed, while English databases included PubMed, Web of Science, and Embase. Search terms included keywords such as “patients,” “inpatients,” “e-Health literacy,” “digital health literacy,” “scale,” “questionnaire,” “assessment tool,” and “instrument.” Databased-specific strategies were employed, and citation tracing was used to supplement the search. Studies focused on eHL assessment with patients as primary targeted sample were eligible for inclusion. For duplicate studies, the most recent or complete publication was included. Reviews, conference abstracts, editorial, commentaries, study protocols, and articles without available full text were excluded. After removing duplicates, two reviewers independently screened all titles and abstracts for eligibility, followed by a full-text review. Discrepancies were resolved through weekly discussion iteratively with a third researcher. Key study characteristics, including author, year, country/region, study type, population, sample size, instruments, and item details, were extracted for analysis.

#### Delphi

To refine the item pool and the structure, a Delphi method was used to integrate expertise across multiple fields, ensuring the content validity and robustness of the scale. This iterative process involved 2 rounds of consultation with experts selected based on the following criteria: (1) intermediate or senior technical titles in the health care field, including academic and clinical roles; (2) at least 10 years of professional experience, with strong theoretical knowledge and practical skills; and (3) willingness to participate voluntarily with informed consent and the ability to provide objective and constructive feedback. A total of 18 experts from 12 provincial-level administrative regions in China were invited, representing diverse fields such as hospital information management, smart health, nursing informatics, nursing management, and clinical nursing, to ensure a balanced knowledge structure among the panel [23].

The first-round Delphi questionnaire included an introduction to the study, the initial item pool, and basic information of experts. Experts rated the importance of each item using a 5-point Likert scale (from “very important” to “not important at all”). In the second round, experts evaluated both the importance and relevance of each item, which was rated on a 4-point Likert scale (from “very relevant” to “irrelevant”). Experts were also allowed to suggest modifications, deletions, or additions, with justifications provided in comment sections. Questionnaires were distributed via email or WeChat and collected on May 28, 2023, and July 20, 2023, respectively.

Statistical analyses were performed using SPSS 26.0. Items with the average importance score >3.5, full score rate >20%, and coefficient of variance (CV) <0.25 were retained. Experts’ suggestions were systematically addressed, and feedback was incorporated into subsequent rounds until consensus was reached [24]. The positivity coefficient of experts was assessed by the return rate of questionnaires, with higher return rates reflecting greater engagement [24]. Expert authority was quantified using the authority coefficient (Cr), calculated as the average of the familiarity coefficient (Cs) and judgment coefficient (Ca): Cr=(Cs+Ca)/2. A Cr ≥0.70 was deemed acceptable [25]. Specifically, the familiarity coefficient measures how familiar an expert is with the topic being evaluated, typically rated on a scale (eg, from 0.2 to 1.0), with higher values indicating greater familiarity. The judgment coefficient reflects the basis on which the expert makes their judgments, determined by weighting sources such as theoretical analysis, practical experience, literature references, and intuition. Additionally, the concentration level of experts’ advice is reflected by the average importance score and full score rate, while the coordination degree was assessed using the Kendall W coefficient and CV of item importance score [25].

#### Pilot Survey, Item Analysis, and Selection

A pilot survey was conducted in August 2023 to refine the scale based on item analysis and participants’ feedback on any ambiguous or unclear items. A convenience sample of 100 adult inpatients from a Grade A tertiary hospital in Hunan, China, was recruited based on the following criteria [26]: (1) age 18 years or older; (2) ability to complete the survey independently or with guidance; and (3) informed consent and voluntary participation. Exclusion criteria included the following: (1) mental illness or severe cognitive impairment; (2) acute or critical illness preventing survey completion; and (3) significant visual, auditory, or language impairments.

Item analysis was performed using a combination of statistical methods, including critical value analysis, correlation coefficients, Cronbach α, and exploratory factor analysis (EFA). For critical value analysis, participants were divided into high- and low-scoring groups based on the top and bottom 27% of total scores, and independent samples *t* tests were performed to compare item scores between these groups. The correlation coefficient method was used to examine the relationships between the total score and each dimension, as well as between each item and its corresponding dimension, using Pearson correlation analyses. Internal consistency reliability was assessed by calculating Cronbach α for the total scale and each dimension. The corrected item-total correlation (CITC) examines the correlation between the score of each item and the full scale minus the contribution of that item to the score. Items with CITCs less than 0.400, and whose removal led to a substantial increase in Cronbach α, were considered to potentially reduce the internal consistency of the dimension. EFA was performed using principal component analysis with eigenvalues >1 and cumulative contribution rate >70% for factor extraction. The Kaiser–Meyer–Olkin (KMO) statistic and Bartlett test of sphericity were used to assess sampling adequacy. KMO values >0.80 were deemed suitable for factor analysis [26]. Collectively, items were considered for deletion if they met at least 2 of the following criteria [27]: (1) nonsignificant critical value (*P*≥.05) in independent sample *t* test; (2) Pearson correlation coefficient <0.40 between the item and its corresponding dimension; (3) CITC <0.40 with a notable increase in Cronbach α upon item removal; and (4) factor loadings <0.45 in EFA or cross-loadings with differences <0.20.

### Step 2: Validation of the AIPeHLS

A cross-sectional study was conducted in September 2023 to validate the scale. A randomized cluster sampling approach was used, with an average of 60 adult inpatients (excluding pediatrics) recruited from each of 9 wards in a Grade A tertiary hospital in Hunan, China, resulting in a total of 532 participants. The scales were distributed in hard copies by the first author (XYF) and two trained researchers (JJ, MYL). Confirmatory factor analysis (CFA) was used to test the alignment of the scale structure with theoretical assumptions. Model fit was evaluated using a range of indices, such as the goodness-of-fit index (GFI), with values closer to 1 indicating better construct validity [28]. Convergent validity was assessed using average variance extracted (AVE) and composite reliability (CR), with AVE >0.50 and CR >0.70 considered acceptable. Discriminant validity was confirmed if the square root of AVE exceeded interdimension correlation coefficients [29].

Content validity was assessed using the content validity index (CVI) derived from Delphi ratings for relevance. Item-level CVI (I-CVI) values ≥0.78 and scale-level CVI (S-CVI) values ≥0.90 were considered acceptable [30]. The I-CVI was calculated by dividing the number of experts who scored 3 or 4 by the total number of experts, while the S-CVI was determined by averaging the I-CVIs of all the items [30]. Criterion validity was evaluated by correlating the AIPeHLS with the Chinese version of eHEALS translated by Guo et al, which was validated among 110 high school students with a Cronbach α of .913 [31]. A higher correlation coefficient indicated stronger criterion validity.

Cronbach α and McDonald omega (ω) coefficients were calculated for the entire scale and its dimensions, with values ≥0.80 indicating good reliability [26]. Finally, the scale was divided into 2 halves, and the correlation between subscale scores was computed for evaluating split-half reliability, with coefficients ≥0.80 considered good reliability [26]. A detailed flow diagram for the development and validation process is provided in Multimedia Appendix 1.

### Ethical Considerations

Ethical approval was obtained from the Research Ethics Board of School of Nursing, Central South University, Hunan, China (No. E202373). Informed consent was obtained from all participants enrolled in this study. All patient data were anonymized, with unique IDs assigned to each participant. The data were securely stored in a password-protected database, accessible only to authorized personnel. Patients were informed about data collection and usage and could withdraw at any time without consequences. As a token of appreciation, each participant received a small gift.

## Results

### Item Pool Generation

A comprehensive review of 934 articles related to eHL was conducted, resulting in the inclusion of 19 studies for item pool development, comprising 8 Chinese and 11 English articles (Multimedia Appendix 1). An initial pool of 53 items was generated based on this review.

### Delphi

To refine the item pool, a 2-round Delphi consultation was carried out with 18 experts from 12 tertiary-level general hospitals, 2 higher education institutions, and 1 national academic organization across 12 provincial-level regions, including Henan, Jilin, Gansu, Shandong, Sichuan, Hunan, Guangdong, Shaanxi, Liaoning, Shanghai, Beijing, and Xinjiang Uygur Autonomous Region. The panel consisted of 14 health care professionals and 4 experts in computer science and engineering. All participants held at least a bachelor’s degree, with approximately 90% holding master’s degrees or higher. Furthermore, nearly 90% of the experts were at vice senior or senior professional levels, and 95% had more than 10 years of working experience, ensuring high expertise and credibility in their feedback (Table 1).

The response rates for both rounds of consultation were 100%, demonstrating positive engagement. Expert authority, quantified by the authority coefficient, was 0.864, indicating high reliability of the consultation results. Item importance scores averaged >3.50 across both rounds, with full-score rates exceeding 20% for 90.57% and 100% of items in the first and second rounds, respectively. CVs for 93.22% and 96.08% of items were <0.25 across the 2 rounds, reflecting consensus among experts. The Kendall W coefficients for both rounds were statistically significant (*P*<.001) with values of 0.249 (*χ²*=262.340) and 0.146 (*χ²*=131.317), respectively. Based on expert feedback and statistical evaluation from the first round, we modified 22 items, added 3 new items, removed 11 items, and 8 items were merged appropriately. In the second round, 16 items were modified and 1 item was removed, leading to a finalized pool of 44 items distributed across 6 dimensions for the initial version of APIeHLS (Multimedia Appendix 1).

**Table 1.** Demographic characteristics of the experts (n=18).

Variables	N (%)
Gender	
Male	4 (22.22)
Female	14 (77.78)
Age (years)	
36–45	8 (44.44)
46–60	10 (55.56)
Education level	
Doctor	8 (44.44)
Master	8 (44.44)
Bachelor	2 (11.11)
Professional title	
Senior	11 (61.11)
Vice-senior	5 (27.78)
Intermediate	2 (11.11)
Work experience	
1–10	1 (5.56)
11-20	3 (16.67)
21–30	9 (50.00)
31–40	5 (27.78)
Whether or not a graduate supervisor	
No	3 (16.67)
Doctoral supervisor	6 (33.33)
Master supervisor	9 (50.00)
Professional background	
Nursing	14 (77.78)
Medical information engineering	1 (5.56)
Computer science and technology	3 (16.67)
Work areas	
Nursing management	13 (72.22)
Clinical nursing	9 (50.00)
Nursing informatics	6 (33.33)
Smart health	5 (27.78)
Hospital information management	4 (22.22)

### Pilot Survey, Item Analysis, and Selection

In the critical value analysis, independent sample *t* tests revealed statistically significant differences (*P*<.001) between the high- and low- scoring groups for all items and confirmed their discriminatory power (Table 2). The item-total correlation coefficients for each dimension ranged from 0.843 to 0.943 (traditional literacy), 0.745 to 0.905 (information literacy), 0.895 to 0.936 (media literacy), 0.929 to 0.971 (health literacy), 0.832 to 0.881 (computer literacy), and 0.909 to 0.947 (scientific literacy) (Table 2). The correlation coefficients between each dimension and the total score ranged from 0.685 to 0.848, all exceeding the threshold of 0.400 and demonstrating statistical significance (*P*<.001). The initial scale demonstrated excellent internal consistency, with a total Cronbach α of .959 and subscale values ranging from 0.952 to 0.975. CITCs for all items exceeded 0.400, and the deletion of any item did not significantly improve the Cronbach α, confirming that all items contributed to the overall measurement consistency (Table 2).

**Figure 1.**
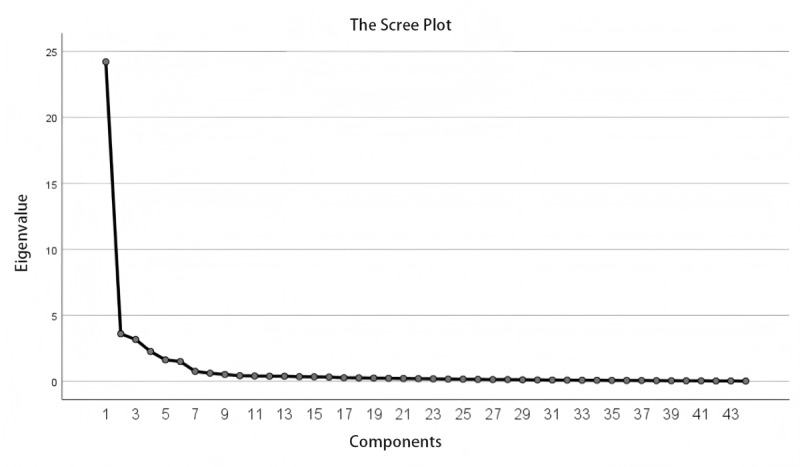
The scree plot.

The KMO value for the initial scale was .921, and the Bartlett test of sphericity yielded a *χ²* value of 5871.995 (*P*<.001), confirming the suitability of the data for factor analysis [26]. Six factors were extracted using the maximum variance method, with eigenvalues of 9.786, 6.668, 6.264, 5.820, 4.252, and 3.562, respectively, accounting for a cumulative variance contribution of 82.616% (Table 3). All factor loadings were ≥0.400, and no cross-loading was observed, supporting the clarity and distinctiveness of the dimensions (Table 4). The scree plot is illustrated in Figure 1. The finalized AIPeHLS included 44 items rated on a 5-point Likert scale from 1 (strongly disagree) to 5 (strongly agree) across 6 dimensions.

**Table 2.** Results of item analysis.

Items	Critical value (t)	Correlation coefficient (r)	CITC^a^	Cronbach α after removement
A1	−14.636	0.932	0.923	0.946
A2	−18.078	0.921	0.881	0.951
A3	−10.850	0.848	0.818	0.957
A4	−12.057	0.843	0.811	0.958
A5	−15.854	0.894	0.874	0.951
A6	−17.298	0.941	0.921	0.946
B7	−8.973	0.824	0.854	0.967
B8	−11.883	0.818	0.853	0.967
B9	−6.407	0.745	0.767	0.969
B10	−8.607	0.781	0.812	0.968
B11	−8.258	0.785	0.808	0.968
B12	−8.892	0.832	0.824	0.968
B13	−6.804	0.774	0.817	0.968
B14	−7.427	0.802	0.855	0.967
B15	−11.125	0.818	0.837	0.968
B16	−7.046	0.819	0.829	0.968
B17	−8.987	0.814	0.842	0.968
B18	−6.709	0.784	0.794	0.969
B19	−10.246	0.905	0.921	0.966
C20	−8.251	0.916	0.897	0.972
C21	−9.884	0.895	0.903	0.971
C22	−6.850	0.904	0.875	0.973
C23	−8.286	0.920	0.920	0.970
C24	−9.067	0.936	0.937	0.969
C25	−9.265	0.918	0.93	0.969
C26	−8.401	0.895	0.892	0.972
D27	−8.439	0.952	0.942	0.972
D28	−8.334	0.971	0.967	0.969
D29	−7.780	0.932	0.923	0.974
D30	−7.796	0.950	0.935	0.972
D31	−7.406	0.908	0.887	0.977
D32	−8.021	0.929	0.899	0.976
E33	−9.244	0.842	0.817	0.959
E34	−8.606	0.870	0.873	0.955
E35	−11.267	0.853	0.871	0.955
E36	−6.631	0.866	0.869	0.955
E37	−6.663	0.844	0.845	0.957
E38	−7.007	0.832	0.824	0.958
E39	−8.683	0.860	0.830	0.957
E40	−11.252	0.881	0.891	0.954
F41	−8.498	0.947	0.913	0.928
F42	−9.156	0.916	0.897	0.933
F43	−7.388	0.909	0.841	0.950
F44	−7.318	0.937	0.883	0.937

a CITC: corrected item-total correlation.

**Table 3.** Results of exploratory factor analysis.

Factors	Eigenvalue			Extraction sums of squared loadings			Rotation sums of squared loadings		
	Total	Contribution rate (%)	Cumulative contribution rate (%)	Total	Contribution rate (%)	Cumulative contribution rate (%)	Total	Contribution rate (%)	Cumulative contribution rate (%)
1	24.215	55.034	55.034	24.215	55.034	55.034	9.786	22.240	22.240
2	3.599	8.179	63.213	3.599	8.179	63.213	6.668	15.154	37.394
3	3.166	7.195	70.408	3.166	7.195	70.408	6.264	14.237	51.631
4	2.256	5.126	75.534	2.256	5.126	75.534	5.820	13.228	64.859
5	1.618	3.677	79.211	1.618	3.677	79.211	4.252	9.663	74.522
6	1.498	3.405	82.616	1.498	3.405	82.616	3.562	8.094	82.616

**Table 4.** Exploratory factor loadings.

Items	Factor loadings					
	Factor 1	Factor 2	Factor 3	Factor 4	Factor 5	Factor 6
A1	0.343	0.288	0.267	0.202	0.761	0.126
A2	0.342	0.274	0.297	0.185	0.693	0.234
A3	0.268	0.362	0.163	0.212	0.685	0.174
A4	0.300	0.307	0.247	0.208	0.633	0.260
A5	0.344	0.342	0.232	0.233	0.683	0.146
A6	0.318	0.300	0.289	0.213	0.733	0.196
B7	0.746	0.281	0.270	0.206	0.220	−0.024
B8	0.770	0.085	0.213	0.216	0.219	0.219
B9	0.732	0.213	0.262	0.115	0.046	0.060
B10	0.746	0.170	0.280	0.123	0.133	0.142
B11	0.716	0.165	0.278	0.102	0.322	0.046
B12	0.759	0.254	0.249	0.082	0.133	0.142
B13	0.759	0.083	0.236	0.254	0.142	0.107
B14	0.816	0.123	0.178	0.182	0.217	0.025
B15	0.702	0.173	0.196	0.345	0.278	0.138
B16	0.813	0.250	0.056	0.181	0.106	0.087
B17	0.741	0.204	0.194	0.202	0.230	0.192
B18	0.765	0.170	0.144	0.204	0.087	0.107
B19	0.838	0.206	0.158	0.228	0.174	0.191
C20	0.250	0.806	0.214	0.093	0.230	0.190
C21	0.247	0.797	0.188	0.159	0.245	0.214
C22	0.193	0.835	0.230	0.080	0.175	0.100
C23	0.221	0.868	0.150	0.108	0.166	0.176
C24	0.215	0.837	0.251	0.156	0.190	0.202
C25	0.262	0.832	0.190	0.167	0.201	0.199
C26	0.231	0.832	0.180	0.189	0.179	0.115
D27	0.277	0.162	0.206	0.850	0.176	0.147
D28	0.243	0.153	0.195	0.886	0.167	0.152
D29	0.260	0.098	0.220	0.857	0.118	0.170
D30	0.248	0.138	0.213	0.868	0.138	0.146
D31	0.273	0.169	0.230	0.798	0.161	0.154
D32	0.248	0.157	0.273	0.790	0.171	0.236
E33	0.307	0.280	0.708	0.127	0.230	0.109
E34	0.297	0.173	0.733	0.305	0.169	0.218
E35	0.365	0.175	0.700	0.266	0.267	0.174
E36	0.216	0.231	0.812	0.151	0.149	0.167
E37	0.231	0.169	0.790	0.246	0.116	0.147
E38	0.238	0.163	0.752	0.219	0.171	0.153
E39	0.249	0.211	0.768	0.158	0.172	0.140
E40	0.399	0.284	0.697	0.287	0.159	0.162
F41	0.192	0.269	0.195	0.225	0.184	0.824
F42	0.193	0.252	0.276	0.273	0.187	0.778
F43	0.148	0.261	0.258	0.191	0.189	0.768
F44	0.197	0.261	0.192	0.251	0.172	0.800

### Validity and Reliability of the APIeHLS

**Figure 2.**
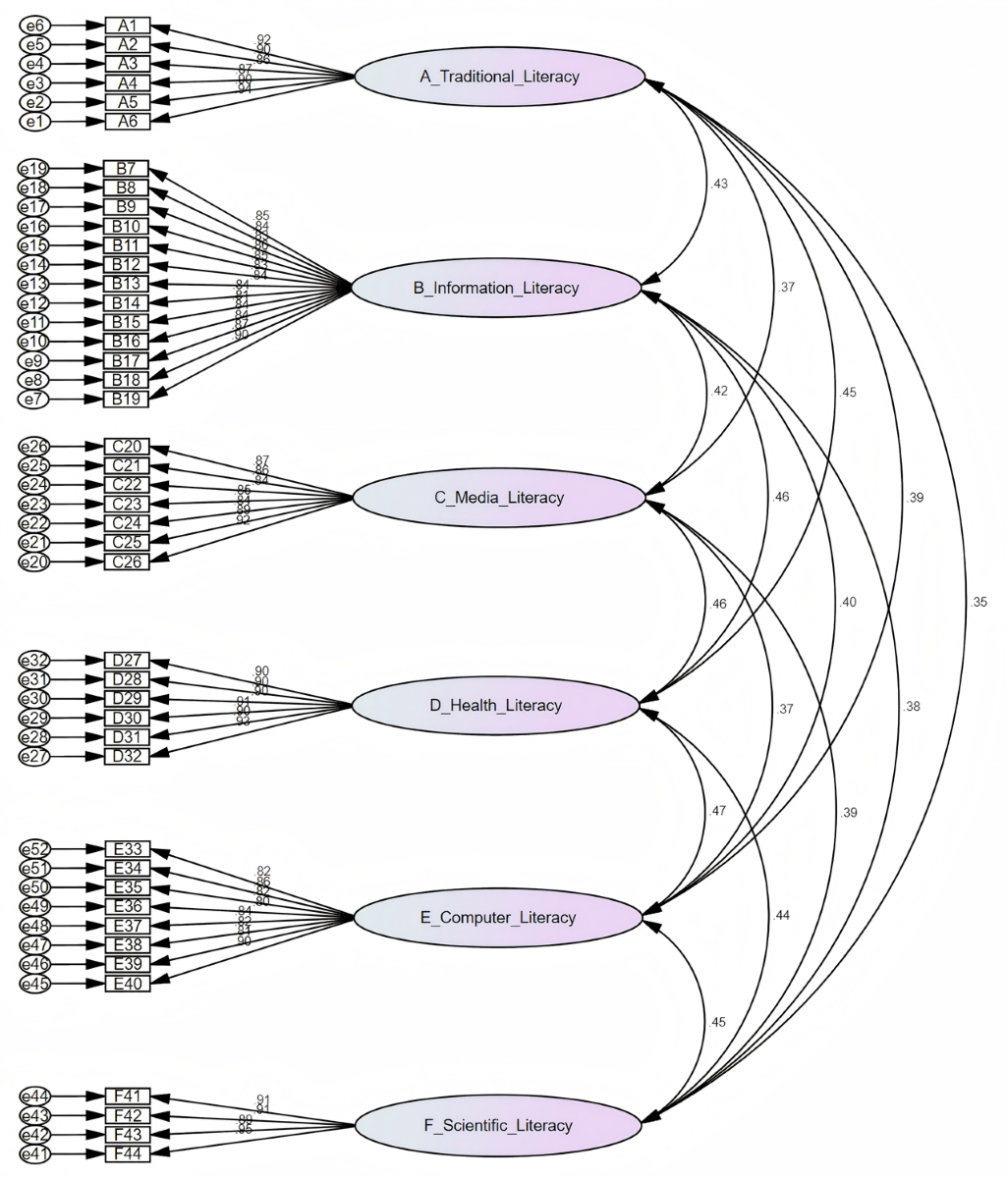
Structural equation modeling for confirmatory factor analysis.

CFA was conducted to validate the factor structure identified in the EFA. Standardized path models were constructed based on data from 532 participants using AMOS 26.0. Fit indices demonstrated acceptable model fit: *χ²*=1974.654 (*df*=887) , GFI=0.854, AGFI=0.837, root mean square error of approximation=0.048, RMR=0.052, comparative fit index=0.957, normed fit index=0.925, and IFI=0.957, which met the criteria of <3.000, >0.900, >0.900, <0.050, <0.050, >0.900, >0.900, and >0.900, respectively. These results confirmed the structural validity of the scale (Figure 2). Convergent validity was supported by standardized factor loadings >0.700, AVE values ranging from 0.695 to 0.835 (>0.500), and CR values between 0.948 and 0.971 (>0.700) (Table 5). Discriminant validity was demonstrated by AVE square roots exceeding interdimensional correlation coefficients, which ranged from 0.354 to 0.466, indicating that each dimension was distinct and unidimensional (Table 6).

**Table 5.** Results of convergent analysis.

Paths	St. estimate	AVE	CR
A. Traditional literacy		0.808	0.962
A6 <--- A	0.944		
A5 <--- A	0.903		
A4 <--- A	0.871		
A3 <--- A	0.859		
A2 <--- A	0.896		
A1 <--- A	0.917		
B. Information literacy		0.718	0.971
B19 <--- B	0.904		
B18 <--- B	0.874		
B17 <--- B	0.84		
B16 <--- B	0.838		
B15 <--- B	0.812		
B14 <--- B	0.84		
B13 <--- B	0.836		
B12 <--- B	0.831		
B11 <--- B	0.852		
B10 <--- B	0.859		
B9 <--- B	0.826		
B8 <--- B	0.845		
B7 <--- B	0.854		
C. Media literacy		0.756	0.956
C26 <--- C	0.923		
C25 <--- C	0.895		
C24 <--- C	0.843		
C23 <--- C	0.852		
C22 <--- C	0.837		
C21 <--- C	0.864		
C20 <--- C	0.867		
D. Health literacy		0.819	0.964
D32 <--- D	0.928		
D31 <--- D	0.9		
D30 <--- D	0.907		
D29 <--- D	0.898		
D28 <--- D	0.898		
D27 <--- D	0.898		
E. Computer literacy		0.695	0.948
E40 <--- E	0.9		
E39 <--- E	0.806		
E38 <--- E	0.824		
E37 <--- E	0.841		
E36 <--- E	0.797		
E35 <--- E	0.817		
E34 <--- E	0.856		
E33 <--- E	0.824		
F. Scientific literacy		0.835	0.953
F44 <--- F	0.946		
F43 <--- F	0.887		
F42 <--- F	0.91		
F41 <--- F	0.911		

**Table 6.** Results of discriminant analysis.

Dimensions	A	B	C	D	E	F
A	0.808	0.426	0.373	0.453	0.385	0.354
B	0.426	0.718	0.421	0.456	0.398	0.380
C	0.373	0.421	0.756	0.459	0.374	0.392
D	0.453	0.456	0.459	0.819	0.466	0.436
E	0.385	0.398	0.374	0.466	0.695	0.452
F	0.354	0.380	0.392	0.436	0.452	0.835
$\sqrt{AVE}$	0.899	0.847	0.869	0.905	0.834	0.914

Content validity, assessed using expert-rated relevance scores, showed excellent results with an S-CVI of 0.961 and I-CVIs ranging from 0.889 to 1.000 (Table 7). Criterion-related validity was evaluated using Spearman rank correlation coefficients, resulting in a total coefficient of 0.992 (All *P*<.001), and subscale correlations ranged from 0.607 to 0.785. This revealed a strong correlation between the AIPeHLS and the external criterion, supporting the scale’s applicability and relevance (Table 8).

The scale demonstrated strong reliability, with a total Cronbach α coefficient of 0.965. The subscales can be used separately, with Cronbach α coefficients of 0.961 (traditional literacy), 0.971 (information literacy), 0.956 (media literacy), 0.964 (health literacy), 0.948 (computer literacy), and 0.953 (scientific literacy). Also, the omega coefficient for the total scale was 0.962, with subscale values ranging from 0.948 to 0.971, indicating high internal consistency. Split-half reliability, assessed using the Spearman–Brown correlation method, yielded a total reliability coefficient of 0.791, with subscale values consistently above 0.960. The final version of the AIPeHLS is available in Multimedia Appendix 2.

**Table 7.** Results of content validity.

Items	Number of experts who rated 3 or 4 (n=18)	I-CVI
A1	18	1.000
A2	18	1.000
A3	18	1.000
A4	18	1.000
A5	18	1.000
A6	18	1.000
B7	18	1.000
B8	18	1.000
B9	17	0.944
B10	17	0.944
B11	17	0.944
B12	17	0.944
B13	18	1.000
B14	18	1.000
B15	18	1.000
B16	18	1.000
B17	18	1.000
B18	17	0.944
B19	16	0.889
C20	17	0.944
C21	17	0.944
C22	17	0.944
C23	16	0.889
C24	18	1.000
C25	18	1.000
C26	16	0.889
D27	18	1.000
D28	18	1.000
D29	18	1.000
D30	18	1.000
D31	18	1.000
D32	17	0.944
E33	16	0.889
E34	17	0.944
E35	16	0.889
E36	16	0.889
E37	18	1.000
E38	17	0.944
E39	17	0.944
E40	16	0.889
F41	18	1.000
F42	16	0.889
F43	18	1.000
F44	17	0.944

**Table 8.** Results of criterion-related validity analysis.

Dimensions	Correlation coefficients
A. Traditional literacy	0.640
B. Information literacy	0.785
C. Media literacy	0.654
D. Health literacy	0.714
E. Computer literacy	0.658
F. Scientific literacy	0.607
Total	0.992

## Discussion

### Principal Findings

This study successfully developed and validated the AIPeHLS, a comprehensive and psychometrically robust instrument tailored to measure eHL in adult inpatients. Grounded in the Lily model, the AIPeHL comprises 44 items that reflect the diverse and evolving competencies required to navigate the digital health landscape, spanning from Web 1.0 to Web 3.0. These items take advantages over existing eHL tools [17-19] by incorporating advanced competencies such as data security, technical problem-solving, and personalization, which are increasingly essential in health care systems that become more digitized and patient-centric. The validation process of AIPeHLS adhered to rigorous psychometric standards to ensure its reliability, validity, and applicability across clinical and research settings. Importantly, the AIPeHLS represents an innovative and forward-looking solution tailored to hospitalized adult inpatients, addressing a critical gap left by previous tools that were either too broad for the general public or targeted at digitally adept younger audiences. This study provides insights for health care providers to better understand how patients make health-related decisions based on their eHealth competencies and develop targeted interventions, while researchers can use the scale to explore opportunities for optimizing user-centered health care solutions.

### Step 1: Development of the AIPeHLS

The development of the AIPeHLS was guided by the Lily model, which conceptualizes eHL as comprising 6 interrelated dimensions. This framework informed the item generation, ensuring that the scale captures both foundational and advanced eHL skills relevant to the digital health challenges faced by inpatients. In this study, a comprehensive literature review and expert consultations were conducted to enhance the relevance and inclusivity of the items.

Within the Lily model, traditional (A), information (B), and media literacy (C) are categorized as analytic components that are foundational and applicable across contexts [1]. For traditional literacy, in addition to a general emphasis on reading and writing (A1, A3) [22], we included more real-world scenarios, such as using voice interactions in instant messaging apps (A2) [21], articulating health concerns (A5) [22], and sharing experiences with peers and caregivers in person or via online health communities (OHCs) (A6) [21]. These items are necessary to understand patients’ engagement in digital health environments and to identify potential barriers to interaction. Numeracy, a critical component of traditional literacy but often overlooked in previous eHL assessments, was also given particular emphasis in this study (A4) as it is essential for inpatients to interpret medication dosages, understand cost-related information, and manage complex treatment regimens. Information literacy refers to the ability to search for (B7, B8), filter (B16), and evaluate (B10, B11, B12, B13) health-related information proficiently [17-22]. The AIPeHLS expands this definition to account for emerging concerns unique to the digital age, such as identifying commercial biases in online content (B15) [21,22], assessing data privacy risks (B17, B19), and managing personal account security (B18). These competencies are increasingly relevant as AI technologies gain prominence in health care, with the percentage of AI-generated information projected to increase from 1% of all human data in 2022 to 10% by 2025 [32]. Despite breakthroughs in the medical field, unsupervised AI tools can potentially generate hallucinations that impact patient decision-making and even lead to unintended negative consequences, such as biased treatment recommendations and inappropriate mental health advice [33]. To mitigate these risks, the scale also evaluates patients’ ability to find or verify online information through consultations with health care professionals (B9, B14) [18], who remain the most trusted sources for validating the credibility and accuracy of health information. This dual focus on independent information evaluation and professional consultation reflects the evolving interaction patterns between patients and health care providers in the digital era. Media literacy is particularly critical in the social media context, where misinformation can spread rapidly, as seen during the pandemic. In this dimension, the AIPeHLS assesses patients’ ability to critically appraise, question, and correct misinformation encountered online (C20, C21, C22). We also considered behaviors related to ethical content sharing, such as posting illness diaries (C23) [20], avoiding spreading unverified content (C24, C25) [21] and preventing plagiarism (C26) [21], providing insights into patients’ roles as both consumers and disseminators of digital health information.

In contrast, health (D), computer (E), and scientific literacy (F) are categorized as context-specific components that rely on more situation-specific skills [1]. Specifically, health literacy empowers patients to use eHealth tools to address health-related issues promptly. Therefore, knowledge of medical terminology (D27) [19], treatment options (D29), and awareness of changes in health conditions (D28) were considered in this dimension. It also evaluates patients’ ability to leverage eHealth tools for decision-making (D31, D32) [22] and self-management (D30) [21], aligning with the goals of eHealth interventions. Computer literacy involves the technical skills required to operate digital devices and navigate innovative tools. Research showed that a lack of experience in using technologies could limit patients’ ability to engage with and benefit from digital health services [34]. The AIPeHLS addresses this gap by assessing familiarity with technological terms (E33), basic operations (E34) [22], safety measures (E35), and problem solving (E36). Moreover, this scale assesses the ability to select (E38), use (E37), and adapt to eHealth tools (E40) [19] for proactive health promotion, such as tracking medical reports, monitoring lifestyle factors (eg, sleep, exercise, and nutrition), and formulating health plans to achieve specific goals [21]. Finally, scientific literacy plays a crucial role in enabling patients to comprehend the scientific foundations underlying health recommendations and critically evaluate the credibility of eHealth tools. For individuals without a background in scientific education, interpreting research-based health information presented online can be particularly challenging [1]. In this study, we assessed whether patients understand that suggestions from eHealth tools are based on time-sensitive research findings (F41, F42) and whether they can recognize the functionalities and limitations of these tools (F43, F44). This focus is novel and significant for selecting eHealth tools rationally and objectively, empowering patients to navigate the complexities of digital health with confidence and competence.

### Step 2: Validation of the AIPeHLS

The construct validity of the AIPeHLS was determined through both EFA and CFA. The EFA results demonstrated a clear factor structure, with each item loading strongly onto its respective dimension and minimal cross-loadings. Subsequent CFA confirmed the factorial composition of AIPeHLS, with commonly used fit indices, such as *χ²*, root mean square error of approximation, comparative fit index, normed fit index, and IFI, meeting or exceeding recommended thresholds. Convergent and discriminant validity were confirmed by the AVE, with values above 0.500 and its square root values greater than interdimensional correlation coefficients. The content validity of the APIeHLS was ensured through a rigorous item development process, with expert panels evaluating the importance, relevance, clarity, and representativeness of each item, achieving CVI values exceeding 0.800 across all dimensions. Criterion-related validity was supported by its high correlation with the Chinese version of eHEALS, a widely accepted instrument, although it has some limitations in capturing the most authentic eHL performance among patients. Regarding reliability, the Cronbach α and McDonald omega estimations showed adequate internal consistency reliability for APIeHLS and its dimensions, and the split-half reliability yielded similarly strong coefficients, with values all above 0.700.

The AIPeHLS was found to be a reliable and valid instrument for assessing eHL in adult inpatients. The inclusion of adult inpatients from various specialties during the development and validation process ensures that the tool is grounded in real-world experiences and needs. Its comprehensive framework and strong psychometric properties make it an effective tool for health care providers to understand patients’ digital health competencies and tailor interventions accordingly. For researchers, our findings provided opportunities to explore the relationship between eHL and health outcomes. Notably, this study represents a significant advancement in eHL measurement by involving the latest technology usage behaviors, offering valuable insights into the development of more effective eHealth interventions and policies.

### Limitations, Strengths, and Future Directions

It is noted that this study has some limitations to be addressed in future research. First, criterion-related validity was evaluated using the Chinese version of the eHEALS, which is often regarded as the gold standard for measuring eHL; however, its ability to accurately reflect the true level of eHL within the target population was significantly constrained by its overly concise and outdated items. Second, the representativeness of results might be limited due to the study sample selected exclusively from a single clinical institution in China. Third, the absence of test-retest reliability due to the relatively short periods of hospital stays may affect the stability of the instrument over time. Accordingly, multicenter studies across diverse populations and time points are expected to further evaluate the measurement invariance and longitudinal reliability of AIPeHLS. Despite these limitations, this study addressed a critical gap in the literature as no prior measure has specifically focused on eHL assessments among inpatient populations who must navigate increasingly complex eHealth challenges. This work represented an innovative advancement in understanding and measuring eHL, particularly by integrating the evolving Web3.0 context and the rapidly advancing eHealth technologies worldwide. Moving forward, the relationships between eHL, as measured by the AIPeHLS, and a range of potential health-related variables could be systematically explored. Furthermore, this scale can be incorporated into studies evaluating the effectiveness of digital health interventions in inpatient settings, providing valuable insights into the impacts of eHealth tools on the health outcomes, self-management capabilities, and overall well-being of hospitalized individuals.

### Conclusions

A psychometrically robust, multidimensional instrument termed the AIPeHLS was developed and validated in this study, comprising 44 items that comprehensively cover all 6 dimensions of the theoretically grounded Lily model of eHL. The AIPeHLS demonstrates a substantial potential to serve as a reliable and valid means of measuring eHL among adult inpatient populations in the evolving Web 3.0 context, empowering health care providers to better understand and improve eHL of inpatients. Furthermore, the deployment of the AIPeHLS may facilitate researchers, engineers, and healthcare providers in evaluating and implementing effective eHealth interventions across diverse clinical settings.

## Supplementary material

10.2196/75657 Multimedia Appendix 1 Flow diagram for the development and validation of the AIPeHLS.

10.2196/75657 Multimedia Appendix 2 The Adult Inpatient eHealth Literacy Scale (AIPeHLS).

## Abbreviations

AI: artificial intelligence
AVE: average variance extracted
CFA: confirmatory factor analysis
CITC: corrected item-total correlation
CR: composite reliability
CV: coefficient of variance
CVI: content validity index
e-HLS: the Electronic Health Literacy Scale
EFA: exploratory factor analysis
eHEALS: the eHealth Literacy Scale
eHealth: electronic health
eHL: eHealth literacy
eHLQ: the eHealth Literacy Questionnaire
GFI: goodness-of-fit index
ICT: information and communications technology
KMO: the Kaiser–Meyer–Olkin
OHCs: online health communities
TeHLI: the Transactional eHealth Literacy Instrument
