# Supplementary material for: The Adult Inpatient eHealth Literacy Scale (AIPeHLS): Development and Validation Study
Source: J Med Internet Res. 2025 Oct 14;27:e75657. doi: 10.2196/75657 (PMC12520623; doi:10.2196/75657)
Supplement: Multimedia Appendix 1 [file jmir-v27-e75657-s001.docx]

**The flow diagram for the development and validation of the AIPeHLS**

- Databases (n = 7): CNKI, Wanfang, VIP, SinoMed, PubMed, Web of Science, Embase
- Search items: 'patients', 'inpatients', 'e-Health literacy', 'digital health literacy', 'scale', 'questionnaire', 'assessment tool', and 'instrument'
- Time period: 1 January 2013 to 10 April 2023
- Included studies (n = 15/934): 8 Chinese [1-8] and 11 English research articles [9-19]

**Item pool generation**

Items modified (n = 22)

Items added (n = 3)

Items removed (n = 11)

The first round of consultation (n = 53)

**Delphi**

The second round of consultation (n = 45)

Items modified (n = 16)

Items removed (n = 1)

**Development**

The initial version of the AIPeHLS (n =44)

- Participants: Adult inpatients
- Sample size: n = 100
- Sampling: Convenience sample
- Setting: Clinical
- Time period: August 2023

**Pilot survey**

The final version of the AIPeHLS (n =44)

Item analysis and selection

- Critical value analysis
- Correlation coefficients
- Cronbach's α
- Exploratory factor analysis

**Item analysis**

- Participants: Adult inpatients
- Sample size: n = 532
- Sampling: Randomized cluster sample
- Setting: Clinical
- Time period: September 2023

**Cross-sectional study**

Reliability of the AIPeHLS

- Cronbach's α
- McDonald's Omega
- Split-half reliability

Validity of the AIPeHLS

- Content validity
- Criterion-related validity
- Confirmatory factor analysis
- Convergent validity
- Discriminant validity

**Validation**

**Validity and Reliability**

**References**

1. Zhang W, Ye F, Zhu LL, Liu XY, Wei JH, Ren L, et al. Development and Reliability and Validity of Hypertension Patients' Health Information Literacy Scale. Pract J Card Cereb Pneum Vasc Dis. 2023;31(5):96-100. [doi: 10.12114/j.issn.1008-5971.2023.00.052]
2. Wang CL. Correlation between electronic health literacy and self-management behaviour in pregnant women with diabetes. China Medical University. 2023.[doi: 10.27652/d.cnki.gzyku.2023.000888]
3. Tang YP, Zhang JY, Zhou MM, Feng SF, Gao F, Shi L. The status quo and influencing factors of digital health literacy in patients with rheumatoid arthritis. Chin Med Pharm. 2023;13(6):16-19,29. [doi: 10.3969/j.issn.2095-0616.2023.06.006]
4. Wei JH. Canonical correlation between health information literacy and self-management ability of patients with hypertension. Jiangsu University. 2022. [doi: 10.27170/d.cnki.gjsuu.2022.000215]
5. Sun YL, Li HW, Sun XN, Li X, Zhu XL. Quality of life in patients with breast cancer combined with type 2 diabetes mellitus and its influencing factors. J Shenyang Med Coll. 2022;24(6):619-624. [doi: 10.16753/j.cnki.1008-2344.2022.06.014]
6. Qian GA, Wu D, Mi Y, Qian JP. The Mediating Role of Perceived Control in the Relationship between Health Information Literacy and Health Information Acquisition Behavior in Stroke Patients. J Clin Nurs. 2022;21(5):2-5. [doi: 10.3969/j.issn.1671-8933.2022.05.001]
7. Liu HY, Tang F, Wang YK, Wei FY, Deng LL. Development and reliability and validity test of health information literacy questionnaire for chronic kidney disease. Chin Nurs Res. 2020;34(24):4362-4367. [doi: 10.12102/j.issn.1009-6493.2020.24.009]
8. Liang CW, Lan CM, Chen MZ. The TCM health information literacy level and its influencing factors among inpatients [J]. J Guangxi Univ Chin Med. 2020;23(1):117-120. [doi: 10.3969/j.issn.2095-4441.2020.01.038]
9. He Y, Guo L, Zauszniewski JA, Wei M, Zhang G, Lei X, et al. A reliability and validity study of the electronic health literacy scale among stroke patients in China. Top Stroke Rehabil. 2023;30(3):272-280. [doi: 10.1080/10749357.2021.2016100] [Medline: 34927574]
10. Chen YC, Cheng C, Osborne RH, Kayser L, Liu CY, Chang LC. Validity testing and cultural adaptation of the eHealth Literacy Questionnaire (eHLQ) among people with chronic diseases in Taiwan: Mixed methods study. J Med Internet Res. 2022;24(1):e32855. [doi: 10.2196/32855] [PMID: 35044310]
11. Scherrenberg M, Falter M, Kaihara T, Xu L, van Leunen M, Kemps H, et al. Development and Internal Validation of the Digital Health Readiness Questionnaire: Prospective Single-Center Survey Study. J Med Internet Res. 2023;25:e41615. [doi: 10.2196/41615] [PMID: 36897627]
12. Lee EH, Lee YW, Lee KW, Kim HJ, Hong S, Kim SH, et al. Development and psychometric evaluation of a new brief scale to measure eHealth literacy in people with type 2 diabetes. BMC Nurs. 2022;21(1):297. [doi: 10.1186/s12912-022-01062-2] [PMID: 36333750]
13. Yoon J, Lee M, Ahn JS, Oh D, Shin SY, Chang YJ, et al. Development and Validation of Digital Health Technology Literacy Assessment Questionnaire. J Med Syst. 2022;46(2):13. [doi: 10.1007/s10916-022-01800-8] [PMID: 35072816]
14. Dang LT, Luong TC, Nguyen DH, Hoang TA, Nguyen HT, Nguyen HC, et al. The Associations of Suspected COVID-19 Symptoms with Anxiety and Depression as Modified by Hemodialysis Dietary Knowledge: A Multi-Dialysis Center Study. Nutrients. 2022;14(12):2364. [doi: 10.3390/nu14122364] [Medline: 35745093]
15. Vitolo M, Ziveri V, Gozzi G, Busi C, Imberti JF, Bonini N, et al. DIGItal Health Literacy after COVID-19 Outbreak among Frail and Non-Frail Cardiology Patients: The DIGI-COVID Study. J Pers Med. 2022;13(1):99. [doi: 10.3390/jpm13010099] [Medline: 36675760]
16. Safdari R, Yu P, Khenarinezhad S, Ghazanfari Savadkoohi E, Javanmard Z, et al. Validity and reliability of the Persian version of the Patient readiness to engage in health information technology (PRE-HIT) instrument. BMC Prim Care. 2022;23(1):50. [doi: 10.1186/s12875-022-01665-3] [Medline: 35305567]
17. Paige SR, Stellefson M, Krieger JL, Miller MD, Cheong J, Anderson-Lewis C. Transactional eHealth Literacy: Developing and Testing a Multi-Dimensional Instrument. J Health Commun. 2019;24(10):737-748. [doi: 10.1080/10810730.2019.1666940] [Medline: 31583963]
18. Karnoe A, Furstrand D, Christensen KB, Norgaard O, Kayser L. Assessing competencies needed to engage with digital health services: Development of the eHealth literacy assessment toolkit. J Med Internet Res. 2018;20(5):e178. [doi: 10.2196/jmir.8347] [Medline: 29748163]
19. Petrič G, Atanasova S, Kamin T. Ill Literates or Illiterates? Investigating the eHealth Literacy of Users of Online Health Communities. J Med Internet Res. 2017;19(10):e331. [doi: 10.2196/jmir.7372] [Medline: 28978496]
