# Supplementary material for: The Adult Inpatient eHealth Literacy Scale (AIPeHLS): Development and Validation Study
Source: J Med Internet Res. 2025 Oct 14;27:e75657. doi: 10.2196/75657 (PMC12520623; doi:10.2196/75657)
Supplement: Multimedia Appendix 2 [file jmir-v27-e75657-s002.doc]

The Adult Inpatient eHealth Literacy Scale (AIPeHLS)

Instructions: The following statements are designed to assess your eHealth literacy. Please select the answer that best reflects your situation.

| Dimensions | Items | Strongly disagree | Disagree | Uncertain | Agree | Strongly agree |
| --- | --- | --- | --- | --- | --- | --- |
| A. Traditional literacy | A1. I can understand the text and images provided by eHealth tools (e.g., hospital official accounts, healthcare apps) on topics such as inpatient guidance and health education. |  |  |  |  |  |
|  | A2. I can use the voice features of eHealth tools (e.g., WeChat voice messaging). |  |  |  |  |  |
|  | A3. When using the input features of eHealth tools, I can accurately write or spell in Chinese (e.g., typing, handwriting, or signing forms). |  |  |  |  |  |
|  | A4. I can, or with the help of tools, perform basic mathematical operations (e.g., addition, subtraction, multiplication, division) to verify hospital expenses or medication dosages. |  |  |  |  |  |
|  | A5. I can use eHealth tools to clearly express my health concerns, worries, and needs to healthcare providers, ensuring they understand my thoughts and feelings. |  |  |  |  |  |
|  | A6. I can use eHealth tools to discuss health issues, share feelings, and exchange experiences with other patients or family members. |  |  |  |  |  |
| B. Information literacy | B7. I know where to find useful health information online. |  |  |  |  |  |
|  | B8. When using eHealth tools, I know what search terms to use to find information relevant to my health needs. |  |  |  |  |  |
|  | B9. I proactively ask healthcare providers how to use eHealth tools to find information related to my health issues. |  |  |  |  |  |
|  | B10. When using eHealth tools, I consider whether the information provided on a health topic is comprehensive. |  |  |  |  |  |
|  | B11. When using eHealth tools, I check the qualifications of the company or publisher (e.g., whether it is an official source), and contact information (e.g., address or phone number) to determine if the information is trustworthy. |  |  |  |  |  |
|  | B12. When using eHealth tools, I check if the information provided is up-to-date or has a publication date. |  |  |  |  |  |
|  | B13. When using eHealth tools, I compare and evaluate differences and connections between various information sources. |  |  |  |  |  |
|  | B14. When using eHealth tools, I verify the authenticity of the information obtained by consulting other sources (e.g., healthcare providers, books, newspapers). |  |  |  |  |  |
|  | B15. When using eHealth tools, I consider if the information provided serves a commercial purpose (e.g., selling medical device or health products). |  |  |  |  |  |
|  | B16. When using eHealth tools, I filter information that meets my own needs. |  |  |  |  |  |
|  | B17. When using eHealth tools, I assess whether uploading personal information (e.g., name, address, health status) to websites is safe. |  |  |  |  |  |
|  | B18. When using eHealth tools, I set privacy permissions (e.g., personal information visible only to designated people or locking bookmarks). |  |  |  |  |  |
|  | B19. I proactively ask and monitor healthcare providers to ensure that personal and medical information obtained through eHealth tools (e.g., personal digital assistants, mobile nursing carts) is protected. |  |  |  |  |  |
| C. Media literacy | C20. I can obtain meaningful information (e.g., about appointments, hospitalization, diseases) from media sources (e.g., news, radio, newspapers). |  |  |  |  |  |
|  | C21. I critically evaluate, analyze, and question information about appointments, hospitalization, or diseases published by the media. |  |  |  |  |  |
|  | C22. I can identify and objectively assess different viewpoints and biases across media sources regarding appointments, hospitalization, or diseases. |  |  |  |  |  |
|  | C23. I know how to share or publish content (e.g., health knowledge, treatment experiences, illness diaries, fundraising appeals) through media and ask healthcare providers for advice. |  |  |  |  |  |
|  | C24. I refrain from spreading unverified information (e.g., monkeypox). |  |  |  |  |  |
|  | C25. I dissuade others from spreading false information (e.g., monkeypox). |  |  |  |  |  |
|  | C26. I do not plagiarize or copy others' texts and images. |  |  |  |  |  |
| D. Health literacy | D27. I understand medical terms and basic knowledge related to my diseases (e.g., disease names, causes, clinical manifestations). |  |  |  |  |  |
|  | D28. I know when to use eHealth tools to obtain information about changes in my health (e.g., worsening or improvement of symptoms). |  |  |  |  |  |
|  | D29. I proactively use eHealth tools to stay informed about advancements in disease treatment options. |  |  |  |  |  |
|  | D30. I can follow health guidance provided by eHealth tools to perform simple self-care or health management (e.g., taking medication on time, getting out of bed, controlling diet). |  |  |  |  |  |
|  | D31. I can use health guidance provided by eHealth tools to answer health-related questions and make health decisions (e.g., diet, medication, or seeking healthcare advice). |  |  |  |  |  |
|  | D32. I can assess whether actions suggested by eHealth tools are effective or problematic and adjust them with healthcare providers' input. |  |  |  |  |  |
| E. Computer literacy | E33. I know basic terms related to information technology (e.g., software, keyboard, links). |  |  |  |  |  |
|  | E34. I can access and perform basic operations on electronic devices (e.g., computers, phones, tablets) in daily life (e.g., clicking a mouse, connecting to the internet, installing software, playing audio or video). |  |  |  |  |  |
|  | E35. I know how to maintain the security of electronic devices (e.g., detecting and removing viruses, blocking malicious software). |  |  |  |  |  |
|  | E36. I can identify and handle common issues with electronic devices (e.g., clearing memory, fixing internet issues). |  |  |  |  |  |
|  | E37. I know how to use eHealth tools to track my lifestyle and health information (e.g., test results, sleep monitoring, exercise frequency, body fat percentage). |  |  |  |  |  |
|  | E38. I know which eHealth tools to choose to meet my health needs and use them to promote my health (e.g., setting health reminders, creating weight loss plans). |  |  |  |  |  |
|  | E39. I know how to personalize eHealth tools to meet my health needs. |  |  |  |  |  |
|  | E40. I can adapt to using different or new eHealth tools. |  |  |  |  |  |
| F. Scientific literacy | F41. I understand the process and methods of scientific research and know that the information provided by eHealth tools (e.g., advice on medication, diet, exercise) is systematically created and scientific. |  |  |  |  |  |
|  | F42. I proactively follow the latest research findings related to my diseases. |  |  |  |  |  |
|  | F43. I know that eHealth tools can help me improve my health. |  |  |  |  |  |
|  | F44. I understand that eHealth tools have timeliness and limitations, and they are updated with scientific progress. Therefore, I choose and use eHealth tools rationally and objectively. |  |  |  |  |  |
